# Accidental Chronic Poisoning with Methotrexate; Report of Two Cases

**Published:** 2017-05-21

**Authors:** Reza Bidaki, Mojgan Kian, Hamid Owliaey, Mojtaba Babaei Zarch, Masoud Feysal

**Affiliations:** 1 Research Center of Addiction and Behavioral Sciences, Shahid Sadoughi University of Medical Sciences, Yazd, Iran.; 2 Diabetes Research Center, Shahid Sadoughi University of Medical Sciences, Yazd, Iran.; 3 Department of Psychiatry, Shahid Sadoughi University of Medical Sciences, Research Center of Addiction and Behavioral Sciences, Yazd, Iran.; 4 Fellowship of toxicity, Assistant professor of Islamic Azad university of Yazd, Yazd, Iran.; 5 Student Research Committee, Shahid Sadoughi University of Medical Sciences, Yazd, Iran.; 6 School of Medicine. Shahid Sadoughi University of Medical sciences, Yazd, Iran.

**Keywords:** Methotrexate, skin ulcer, toxicity, poisoning, case reports, emergency treatment

## Abstract

Methotrexate has been used widely in dermatology, oncology and rheumatology fields. However, methotrexate-induced mucocutaneous lesions may occur in rare cases. In this case presentation, we report two cases of accidental poisoning with methotrexate. They had accidentally used methotrexate instead of digoxin. This case report emphasizes that early diagnosis and appropriate management is critical in order to improve outcome.

## Introduction

Methotrexate (MTX) is a systemic immunosuppressive agent that was introduced in the 1950s (1, 2). It is a folate antimetabolite that binds to an enzyme named dihydrofolate reductase, which ultimately leads to inhibition of DNA synthesis (3, 4). MTX is a drug used in treatment of various malignancies, early ectopic pregnancy or chronic inflammatory diseases such as some types of carcinoma, rheumatoid arthritis, psoriasis and etc. (5, 6). Accidental poisoning with MTX is not a common condition and was rarely reported in the literature (7). Most of our knowledge regarding MTX poisoning were derived from serious adverse reactions at therapeutic doses or reports about acute oral overdose (8). Unlike previously reported cases, here we report two cases of accidental chronic poisoning with MTX. 

## Case report

***Case 1***

The patient was an 88-year-old woman. She was admitted to the emergency department with weakness as main chief complaint. She had dyspnea, gastrointestinal symptoms such as nausea and vomiting, abdominal pain, dyspepsia, pruritus, epistaxis, and mouth sores from 3 days ago. The symptoms were progressive. 

In evaluation of consumed drugs it was determined that she has been continuously taking MTX for about 5 months. In search for the cause of taking MTX and checking the prescriptions, it was revealed that the drug store had mistakenly given the patient MTX instead of digoxin.

She had used MTX 1.25 mg for 5 consecutive months and 2.5 mg five days a week in the last month. On admission, her vital signs were as follows: Blood Pressure = 90/60 mmHg, Pulse Rate = 82/minutes, Respiratory Rate = 12/minutes and Temperature = 38.2°C. 

**Figure 1:**
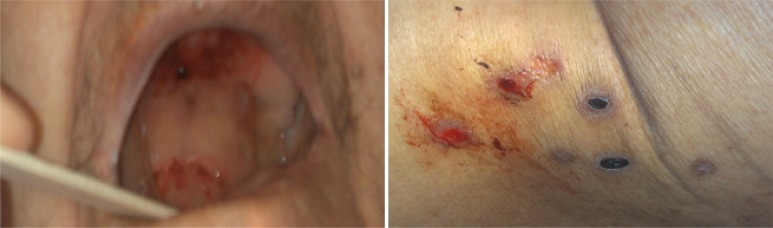
Mucocutaneous lesions in case number 1

On physical examination, generalized erythema involving back and leg, face edema, mucositis, stomatitis and difficulty in gait were apparent. Examination of other organs did not reveal any positive findings. Figure 1 shows her mucocutaneous lesions at presentation.

Laboratory test results on admission were as follows: Hemoglobin=9.1 g/dl, WBC=3200/mm^3^, Platelet counts = 50000/mm^3^, BUN = 81 mg/dl, creatinine=1.8 mg/dl, AST=18 IU/L, ALT=17 IU/L, PT=14s, PTT=36s, INR=1.14, Bilirubin total=1.2 mg/dl and Bilirubin Direct=0.3 mg/dl. 

She was admitted to intensive care unit (ICU). Platelets, folinic acid (as MTX antidote), antibiotics and granulocyte colony stimulating factor (G-CSF) were administered and dermatology cares were considered for her. Finally, she died due to pulmonary edema resulting from her underlying cardiac disease 4 days after admission.

***Case 2***

The patient was a 68-year-old woman, who was referred to the emergency department because of oral ulcer, limb paresthesia and difficulty initiating movement from one week ago. Muscular force was reduced gradually. The patient didn't have any history of trauma. The patient had accidentally used one MTX tablet each day, instead of Digoxin, since one month ago (due to a mistake in the drug store, she was given MTX instead of digoxin).

In the past medical history, the patient had a history of diabetes mellitus, hypertension, heart failure and hyperlipidemia. Other drugs used by the patient included losartan, aspirin, carvedilol, L-carnitine and triamterene-H. 

On admission, she was alert and her vital signs were as follows: Blood Pressure = 110/70 mmHg, Pulse Rate = 74/minute, Respiratory Rate =16/minute and Temperature = 36.8°C. 

**Figure 2:**
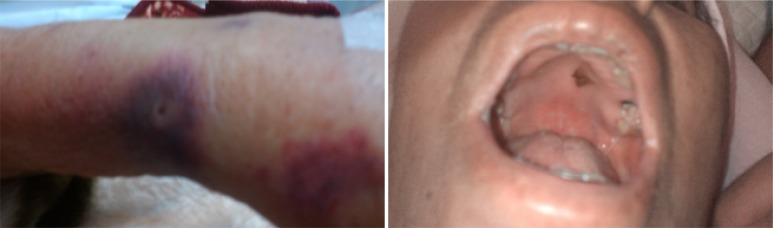
Mucocutaneous lesions in case number 2

On physical examination, level of consciousness was normal. There was no evidence of respiratory distress. Neurological examinations revealed normal cerebellar tests. Upper limb muscular force was normal and lower limb force was 4/5 in proximal and 3/5 in distal. Hemorrhagic ulcers were seen in palate and lips. Figure 2 shows her mucocutaneous lesions at presentation.

Laboratory tests were as follows: Hemoglobin=11.2 g/dl, WBC=10.9/mm^3^, Platelet counts = 20000/mm^3^, Natrium = 140 mEq/l, Potassium = 3.5 mEq/l, urea=36 mg/dl, creatinine=0.85 mg/dl, PT=13 s, PTT=27 s, INR=1.3, ALT=43 IU/L, AST=34 IU/L, ALP=200 IU/L.

Magnetic resonance imaging (MRI) and also electromyogram/nerve conduction velocity (EMG/NCV) tests were normal. Echocardiography revealed ejection fraction (EF) of 20%. With diagnosis of MTX poisoning, the patient underwent treatment with folinic acid for 10 days. The patient was discharged with good general condition and improvement of weakness and lower limb paresthesia 5 days later. 

## Discussion

MTX toxicity is characterized by nausea, vomiting, diarrhea, myelosuppression, pancytopenia, liver dysfunction, acute renal failure (ARF), pulmonary symptoms, mucositis, stomatitis, ulceration/erosion of the gastrointestinal system and cutaneous ulcerations (9-11). Although methotrexate toxicity can cause kidney injury and change the renal function, sometimes renal dysfunction like an acute renal failure can also induce methotrexate toxicity (4, 12-14). However, cutaneous ulceration may be considered as an early clinical sign of imminent systemic toxicity and patients may only present with isolated cutaneous lesions (15-17).

The side effects of MTX may occur through dose dependent or idiosyncratic mechanisms. Interestingly, dose dependent mechanism occurs in bone marrow cells, epidermal cells and epithelial gastrointestinal system. Some factors such as drug interactions and incorrect administration of drugs are triggers for appearance of side effects (9). Simultaneous use of MTX with drugs interacting with it such as proton-pump inhibitors, trimethoprim/sulfamethoxazole, doxycycline, non-steroidal anti-inflammatory drugs (NSAIDs), and salicylates that decrease protein binding or reduce renal clearance, as well as excessive alcohol consumption could play an important role in this regard (18). 

Elevation of serum aminotransferase levels, elevation in serum uric acid, leukopenia, thrombocytopenia, and anemia may be noticed in laboratory tests of MTX poisoned patients (19). Measurement of methotrexate concentration via radioimmunoassay in plasma, serum, or urine samples could lead to definite diagnosis (19). Diagnostic biopsy from ulceration sites is rarely required, but helpful (2).

Withdrawal of MTX and administration of intravenous folinic acid (leucovorin) as early as possible after exposure is the most effective initial treatment that should not be delayed for any reason (2, 19).

Treat persistent nausea and vomiting with several antiemetic agents such as metoclopramide, ondansetron, promethazine, haloperidol, benzodiazepines, or even corticosteroids. Intravenous fluids and urine alkalization via bicarbonate infusion are highly advised. Administration of colony stimulating factors is necessary if severe neutropenia exists. Transfusion of platelets and/or packed red cells may be needed in patients with severe thrombocytopenia, anemia, or hemorrhage. The patient should be closely monitored for signs of bleeding, clinical evidence of infection, abnormalities in serum electrolytes, renal failure and hepatic function. A chest radiograph should be obtained in patients with respiratory symptoms and skin-directed therapy must to be applied (19).

In patients with impaired renal function who develop toxicity and in cases of acute overdose, glucarpidase has been used to rapidly catabolize MTX to an inactive metabolite, aiding its clearance (2, 19, 20). 

Although MTX toxicity can be a fatal poisoning, proper management, early diagnosis and follow-up of the patients in emergency department as well as in ICU can resolve the complications and save the patients’ lives. It seems that computerized prescription system is a promising method to reduce human errors.
